# Phenibut intoxication in a patient on methadone substitution therapy: A case report

**DOI:** 10.1192/j.eurpsy.2023.1830

**Published:** 2023-07-19

**Authors:** K. Ceranic Ninic, A. Celofiga

**Affiliations:** Department for psychiatry, UKC Maribor, Maribor, Slovenia

## Abstract

**Introduction:**

Phenibut is a synthetic γ-aminobutyric acid (GABA) agonist used to treat symptoms of mental disorders like anxiety and insomnia. The substnce is licensed and widely used in Russia. Its recreational use is increasing in many countries as it is easily accessible online. In a recent years, several case reports of phenibut intoxication and withdrawal have been published worldwide.

**Objectives:**

We present a case report of intoxication with phenibut in a patient with psychoactive substances use disorder on regular methadone substitution therapy.

**Methods:**

A 40-year-old male was hospitalized for the first time in the psychiatrc ward due to intoxication with phenibut. Before admission, he was confused, with incoherent speech. Bradypnea and somnolence were observed. A urine screening test was positive for methadone and benzodiazepines, while there was no information on phenibut intake at that time. After receiving naloxone, the patient became, agitated and haloperidol was introduced.

**Results:**

The main symptoms of phenibut intoxication, which have also been described in other cases, are cardiovascular effects, insomnia, anxiety, agitation, hallucinations, and reduced level of consciousness. In our case, a more heterogeneous clinical picture of intoxication was observed, due to the simultaneous use of methadone and other PAS.

**Conclusions:**

The availability of various synthetic substances available online is constantly increasing. Their use is most common in the population with substance use disorders, in patients on substitution therapy and in patients with mental disorders in general. The clinical picture of intoxication can thus be very diverse and atypical, which requires increased attention from clinicians.

**Disclosure of Interest:**

None Declared

